# Engineered organoid platforms for mechanistic studies of gut microbiota-cancer interactions

**DOI:** 10.3389/fbioe.2026.1905559

**Published:** 2026-10-26

**Authors:** Man Sun, Jing-Mian Jiao, Dan Zang, Mei-Xi Lin, Huan Zhou, Jun Chen

**Affiliations:** Department of Oncology, The Second Hospital of Dalian Medical University, Dalian, Liaoning, China

**Keywords:** engineered platforms, gut microbiota, host-microbe co-culture, microfluidic chips, organoids, tumor microenvironment

## Abstract

Associations between the gut microbiota and cancer are increasingly reproducible at the cohort level, but association alone cannot establish whether a microorganism, microbial product, or metabolite directly alters tumor-relevant host biology. Engineered organoid systems address this gap by placing defined microbial exposures against human or genetically specified epithelia under experimentally controlled conditions. The strength of the resulting inference depends on platform architecture. Three-dimensional organoids preserve epithelial organization but restrict luminal access; microinjection and apical-out configurations enable direct microbial exposure; organoid-derived monolayers permit directional sampling and barrier measurements; and microfluidic systems add flow, oxygen gradients, and longer-term host-microbe co-culture. Tumor organoids and defined immune or stromal co-cultures can be added when the biological question requires cancer-specific or multicellular readouts. This review evaluates these platforms according to the mechanistic questions they can answer and distinguishes evidence from live microorganisms, complex communities, cell-free microbial products, purified metabolites, and clinical associations. The strongest organoid-based evidence currently concerns microbial genotoxicity, epithelial transformation, barrier and repair responses, selected tumor-growth phenotypes, and intestinal treatment toxicity. Direct organoid evidence linking the gut microbiota to distant, non-gastrointestinal tumors remains limited despite substantial clinical and animal data. We further discuss how physicochemical control, omics-based benchmarking, and standardized reporting can improve reproducibility without adding unnecessary model complexity. The central requirement is explicit matching of experimental architecture to the causal claim being tested.

## 1 Introduction

Organoids are three-dimensional culture systems generated through the *in vitro* self-organization of tissue stem cells, pluripotent stem cells, or patient-derived tumor cells (Sato et al., 2009; 2011; Spence et al., 2011; Huch et al., 2015; McCauley and Wells, 2017; Almeqdadi et al., 2019; Liu et al., 2025; Wang et al., 2026). They can partially preserve the spatial architecture, cellular lineage composition, and molecular heterogeneity of the tissue of origin. Compared with conventional two-dimensional cultures, organoids more closely recapitulate *in vivo* tissue states and enable the investigation of cellular differentiation, tissue homeostasis, disease progression, and therapeutic responses. These properties have made organoids useful platforms for disease modeling and translational research.

In cancer research, organoids can maintain selected genetic and phenotypic features of tumor cells and provide a tractable system for reconstructing defined components of the tumor microenvironment. Tumor tissues contain malignant epithelial cells, immune cells, fibroblasts, vascular components, extracellular matrix, and diverse soluble factors, and their behavior is shaped by cell-cell interactions, paracrine signaling, and matrix context (de Visser and Joyce, 2023; Rauner et al., 2025). Conventional models rarely preserve tissue architecture, cellular heterogeneity, and microenvironmental dependence simultaneously. Co-culture and engineered culture strategies can therefore be used to model selected interactions rather than to reproduce the entire tumor ecosystem (Asai et al., 2017; Öhlund et al., 2017; Drost and Clevers, 2018; Neal et al., 2018; Mucciolo et al., 2024). Air-liquid interface culture can retain tumor-infiltrating lymphocytes in patient-derived organoids, supporting analysis of immune checkpoint responses (Neal et al., 2018). Tumor organoid co-culture with cancer-associated fibroblasts can similarly resolve direct contact and paracrine signaling (Öhlund et al., 2017; Mucciolo et al., 2024). Patient-derived gastrointestinal tumor organoids also preserve clinically relevant heterogeneity and have been used for drug response assessment (Tiriac et al., 2018; Vlachogiannis et al., 2018; Vazquez-Armendariz and Tata, 2023; Thorel et al., 2024).

The gut microbiota adds a distinct experimental problem because clinical sequencing and animal studies do not readily separate direct microbial effects from systemic host responses. Intestinal microorganisms and their metabolites can regulate inflammation, epithelial barrier integrity, stem cell fate, metabolic pathways, and antitumor immunity (Agus et al., 2018; Helmink et al., 2019; Baas et al., 2024; Dong et al., 2024). Two-dimensional cultures lack tissue architecture, whereas animal models provide integrated physiology but limited control over strain identity, exposure route, host genotype, immune state, and drug conditions. Intestinal organoids, tumor organoids, and microfluidic systems allow selected variables to be manipulated in human or genetically defined tissues (Kasendra et al., 2018; Bozzetti and Senger, 2022; Wang Q. et al., 2024). This review therefore retains a cancer-centered overview of engineered tumor organoids, then focuses on intestinal platforms that test direct host-microbe interactions, microbial products, and metabolites. Clinical and animal evidence is used to establish relevance or define boundaries, not as equivalent organoid evidence. Although the primary focus is the intestinal microbiota, selected gastrointestinal host-microbe organoid systems, including gastric *Helicobacter pylori* models, are included when they provide directly informative mechanistic precedents for microbiota-associated carcinogenesis.

## 2 Applications of organoid models in cancer research

Organoids rely on the intrinsic self-organizing capacity of cells to reconstruct key structural and functional features of the tissue of origin *in vitro*. Advances in co-culture, gene editing, and engineered culture platforms have expanded their application from epithelial expansion to tumor microenvironment reconstruction, drug response assessment, and resistance studies. Current systems nevertheless remain constrained by incomplete immune and vascular components, culture-dependent selection, limited long-term stability, and insufficient standardization (Fatehullah et al., 2016; Drost and Clevers, 2018; Vlachogiannis et al., 2018; Vazquez-Armendariz and Tata, 2023; Mao and Hu, 2024; Li et al., 2026).

### 2.1 Engineered tumor organoid platforms for reconstructing microenvironmental interactions

Tumor organoids are human-relevant three-dimensional models that partially preserve the genetic alterations, histological organization, and functional characteristics of primary tumors. Patient-derived tumor organoids maintain substantial interpatient heterogeneity and provide experimentally accessible systems for investigating tumor evolution and therapeutic responses. Conventional tumor organoids, however, are primarily composed of malignant epithelial cells embedded in extracellular matrix-derived scaffolds and therefore lack many fibroblast, immune, vascular, and microbiota-associated signals. Engineering strategies are most informative when they add a component required by a defined hypothesis rather than increasing complexity without a corresponding readout (Öhlund et al., 2017; Drost and Clevers, 2018; Mao and Hu, 2024).

Engineered tumor organoid platforms include patient-derived tumor organoids, genetically engineered mouse tumor organoids, stromal or immune co-cultures, and organoid-on-chip systems. Human patient-derived organoids are valuable for translational studies because they retain patient-specific genomic characteristics and heterogeneous therapeutic responses, whereas mouse-derived organoids remain useful for dissecting tumor initiation in defined genetic backgrounds (Fatehullah et al., 2016; Drost and Clevers, 2018; Tiriac et al., 2018; Vlachogiannis et al., 2018). These systems should not be treated as interchangeable. Species, tissue source, disease state, and added cellular components determine whether a result supports a human tumor-cell response, a host genetic mechanism, or a multicellular interaction.

Stromal and immune co-cultures illustrate the value of this selective reconstruction. Pancreatic tumor organoids have shown that inflammatory and myofibroblastic cancer-associated fibroblast states are regulated by distinct cytokine programs, with IL-1-driven JAK-STAT signaling opposed by TGF-beta signaling (Öhlund et al., 2017). Air-liquid interface and lymphocyte-organoid co-cultures preserve or reintroduce immune populations and permit measurement of antigen presentation, immune activation, and tumor-cell killing (Neal et al., 2018; Bar-Ephraim et al., 2020; Wang et al., 2025; Najafi et al., 2026). These models can test whether a microbial signal acts primarily on tumor epithelium, stromal cells, or immune cells, but they do not reproduce systemic immune trafficking or whole-body microbial metabolism.

For tumor-gut microbiota studies, the value of engineered tumor organoids lies in reconstructing a selected biological interface. A tumor organoid exposed to a purified metabolite tests a tumor-cell response. A tumor organoid with immune cells can test immune-dependent killing. A compartmentalized chip can test transport across an epithelial barrier. The supported conclusion therefore depends on the tissue source, exposure type, and compartments included in the experiment.

### 2.2 Engineered organoid platforms for anticancer therapy and microbiota-modulated treatment responses

The clinical translation of tumor organoids has primarily focused on predicting therapeutic responses and identifying mechanisms of treatment resistance. Unlike conventional cancer cell lines, which may undergo genetic and transcriptional drift during long-term culture, patient-derived tumor organoids can preserve important molecular features of the original tumor and provide functional platforms for drug evaluation (Ben-David et al., 2018; Tiriac et al., 2018; Vlachogiannis et al., 2018; Thorel et al., 2024). Their current role remains complementary to animal studies and clinical trials.

Patient-derived gastrointestinal tumor organoids represent one of the most clinically advanced applications. Organoids established from metastatic gastrointestinal cancers reproduced heterogeneous responses to anticancer drugs that were associated with clinical outcomes (Vlachogiannis et al., 2018). Pancreatic cancer organoid profiling similarly identified patient-specific chemotherapy responses (Tiriac et al., 2018). These studies principally evaluate tumor-intrinsic determinants of treatment response. They do not by themselves establish that a microbiota-associated clinical signal acts directly on tumor cells.

Microbiota-associated exposures can be added when the experiment is designed around a defined mediator. Gut microbial communities influence anticancer therapy through immune activation, bioactive metabolites, and epithelial barrier regulation (Agus et al., 2018; Gopalakrishnan et al., 2018; Helmink et al., 2019; Ornelas et al., 2022; Dong et al., 2024). A tumor organoid treated with a microbial metabolite can establish concentration-dependent tumor-cell activity, whereas clinical microbiome associations and animal transfer experiments address population relevance and integrated physiology. Evidence from these systems should be interpreted together without merging their causal strength.

Several limitations restrict the clinical application of microbiota-associated tumor organoid platforms. Organoid establishment efficiency, extracellular matrix, culture medium, passage, and microbial compatibility vary between laboratories. Epithelial-only tumor organoids cannot reproduce immune, stromal, and vascular interactions required for immunotherapy studies. Omics methods are readouts for benchmarking molecular fidelity or identifying candidate mechanisms; they are not structural components of an organoid platform (Brazovskaja et al., 2019; Liu et al., 2019; Thorel et al., 2024). These distinctions define what can be concluded from each treatment experiment.

## 3 Engineered intestinal organoid platforms for modeling tumor–gut microbiota interactions

### 3.1 Intestinal organoid platforms for host–microbiota interface reconstruction

Engineered intestinal organoid and organ-on-chip systems reconstruct selected components of the intestinal microenvironment under controlled conditions. Human intestinal organoids preserve multilineage differentiation capacity, epithelial polarity, and key features of intestinal architecture (Kasendra et al., 2018; Bozzetti and Senger, 2022; Baas et al., 2024; Wang Q. et al., 2024). Conventional three-dimensional organoids, apical-out organoids, organoid-derived epithelial monolayers, and microfluidic intestine-on-chip systems differ in epithelial access, microbial viability, oxygen control, sampling, and capacity to incorporate additional cell types.

Architecture determines which microbial exposure is biologically interpretable. The apical surface is enclosed in conventional basal-out organoids, so direct luminal exposure generally requires microinjection (Hill et al., 2017). Apical-out organoids reverse polarity and permit direct short-term contact with microorganisms while retaining a three-dimensional epithelial structure (Co et al., 2019). Organoid-derived monolayers provide directional apical and basolateral access and support barrier resistance, permeability, and transport measurements (Foulke-Abel et al., 2016; Kozuka et al., 2017). Defined hydrogels and scaffold-guided tissues improve control of matrix mechanics and epithelial geometry (Gjorevski et al., 2016; Nikolaev et al., 2020), while organoid-derived intestine-on-chip systems add perfusion, mechanical deformation, and separate luminal and vascular compartments (Kim et al., 2012; Kasendra et al., 2018; Jalili-Firoozinezhad et al., 2019; Shin et al., 2020). Engineered intestinal tissues containing additional neural or mesenchymal components further show that tissue maturity and cellular composition can be adjusted to the question being studied (Workman et al., 2017). No configuration is uniformly superior.

Human primary epithelial organoids can capture donor-dependent responses to microbial stimulation. Bornholdt et al. showed that organoids derived from different individuals exhibited distinct responses to *Bifidobacterium* BB-12 and *Lactobacillus rhamnosus* GG, whereas Caco-2 cultures did not reproduce comparable interindividual variability (Bornholdt et al., 2023). The system used primary human epithelium and defined microbial stimulation, so it supports a donor-specific epithelial response rather than a conclusion about tumor development.

Normal intestinal organoids are relevant when they identify epithelial processes that precede malignant transformation or modify treatment injury. Colorectal tumorigenesis is associated with disruption of barrier integrity, stem cell regulation, inflammatory signaling, and microbial homeostasis. A human colonic organoid-mast cell co-culture showed that fecal supernatant altered serotonin transporter expression through mast cell and prostaglandin E2 signaling (Gao et al., 2022). This was not a cancer model and used a cell-free complex exposure, but it demonstrated an immune-dependent route through which microbial products can modify epithelium.

Immune-derived signals also regulate intestinal stem-cell activity and epithelial regeneration. Human and mouse organoid systems have shown that IL-22 and other lymphocyte-derived signals alter epithelial differentiation and repair (Lindemans et al., 2015; Bar-Ephraim et al., 2020; Fischer et al., 2025; Tsai et al., 2026). *Bacteroides thetaiotaomicron*-associated propionate promoted goblet-cell differentiation through receptor-dependent signaling in intestinal organoids (Wang X. et al., 2024). These experiments identify epithelial or immune mechanisms relevant to carcinogenesis and treatment toxicity, although purified metabolites and cytokines do not reproduce live microbial colonization.

Live microorganisms and cell-free microbial structures should be distinguished. *Lactobacillus reuteri* promoted epithelial regeneration in intestinal organoid models under inflammatory conditions (Wu et al., 2020). Bacteria-derived membrane vesicles induced DNA damage and dysplastic changes in colonic epithelial models (Miyakawa et al., 2024). Host genetic perturbations further showed that epithelial inflammatory and regeneration pathways modify susceptibility to microbial stress (Lu et al., 2021; Cheng et al., 2024). The first type of experiment tests an intact organism, the second tests a cell-free microbial product, and the third tests host susceptibility. Their readouts are complementary but not equivalent.

Together, these studies establish the engineering principles required for tumor-gut microbiota research: controlled apical exposure, defined oxygen conditions, preservation of epithelial polarity, explicit host genotype, and addition of immune components only when the proposed mechanism requires them. They also explain why normal intestinal organoids remain necessary for questions about epithelial initiation, barrier failure, and treatment-related injury.

### 3.2 Microbiota-driven tumor initiation, progression, and therapeutic response in Organoid models

Organoid experiments provide their strongest causal contribution when a defined microbial exposure precedes and produces a tumor-relevant host phenotype. Microbiome sequencing identifies associations, and animal models test integrated physiology, but neither alone isolates a direct human epithelial mechanism (Arthur et al., 2012; Baas et al., 2024; Minot et al., 2024; Yao et al., 2026). Intestinal and tumor organoids can narrow this gap when microbial strain, exposure route, host tissue, and readout are specified.

Genotoxic *Escherichia coli* carrying the *pks* pathogenicity island provides the clearest example. Repeated exposure of human intestinal organoids to colibactin-producing *pks*-positive *E. coli* generated a characteristic mutational signature that was subsequently detected in human colorectal cancer genomes (Pleguezuelos-Manzano et al., 2020; Rosendahl Huber et al., 2021). This experiment directly connects a defined bacterium, a known genotoxin, human intestinal epithelium, and a tumor-associated genomic pattern. It provides stronger causal support than a cross-sectional microbiome association, but it does not reproduce long-term community ecology, diet, inflammation, or immune selection in patients.

*Helicobacter pylori* studies provide a second line of direct host-bacterium evidence. Human pluripotent stem cell-derived gastric organoids and adult gastric epithelial organoids have been infected with *H. pylori*, allowing bacterial localization and epithelial responses to be examined in human tissue (McCracken et al., 2014; Bartfeld et al., 2015). Gastric organoid co-culture has also shown early induction of epithelial PD-L1 and suppression of local T-cell responses (Holokai et al., 2019). Related stromal and host genetic studies demonstrated that R-spondin signaling regulates gastric progenitor expansion and that NOD1 sensing and ATG16L1-dependent autophagy modify inflammatory or carcinogenic responses (Sigal et al., 2017; Suarez et al., 2019; Mommersteeg et al., 2022). These findings are mechanistically related but arise from different epithelial sources and cellular contexts, which should be stated when conclusions are compared.

Microbial signals are not uniformly pro-tumorigenic. TLR5-dependent epithelial signaling can promote recruitment of barrier-supportive macrophages (Tsai et al., 2026). *Lactobacillus gallinarum*, a diet-enriched Coriobacteriaceae strain, and *Lactococcus lactis* HkyuLL 10 have each been linked to colorectal tumor phenotypes through combinations of microbial, organoid, animal, and metabolic experiments (Sugimura et al., 2021; Su et al., 2024; Tang et al., 2024). In several of these studies, organoids were exposed to conditioned medium or an identified metabolite rather than viable bacteria. The exposure should therefore be described as microbial-product or metabolite validation when intact microorganisms were not placed in direct contact with the organoid.

Complex microbial exposure is closer to the ecological question but harder to attribute. Defined communities embedded in gradient hydrogels permit removal or add-back of individual members and have been used to examine microbiome-drug interactions (Zheng et al., 2023). Anaerobic intestine-on-chip systems can maintain complex human microbiota adjacent to living epithelium under controlled oxygen gradients (Jalili-Firoozinezhad et al., 2019). These platforms can test whether a community state is sufficient to alter a host phenotype, but strain-level attribution still requires reduction experiments using defined consortia, isolates, or candidate metabolites.

The present evidence therefore supports different levels of inference. Direct live microorganism-organoid exposure can establish temporal order and a selected host-cell mechanism. Cell-free products and purified metabolites can establish activity of a defined mediator. Animal experiments test integrated physiology, and clinical associations establish human relevance. Conclusions about tumor initiation or treatment response are strongest when these levels converge rather than when one is used as a substitute for another.

The gut microbiota also has substantial clinical and animal evidence in distant tumors, including associations between gut microbial composition and response to PD-1 blockade in melanoma and other epithelial tumors (Gopalakrishnan et al., 2018; Helmink et al., 2019). Direct human organoid reconstruction of the complete gut microbiota-systemic immunity-distant tumor axis, however, remains uncommon. A metabolite-treated distant-tumor organoid establishes a tumor-cell response, and an immune co-culture can establish a defined immune mechanism, but neither alone reconstructs intestinal transport, systemic immune priming, and tumor exposure. This boundary explains why direct organoid evidence remains concentrated in gastrointestinal tissues.

### 3.3 Microbial metabolite validation using engineered Organoid platforms

Engineered intestinal and tumor organoid platforms enable controlled evaluation of microbiota-derived metabolites on epithelial proliferation, stem-cell maintenance, barrier integrity, and tumor-associated signaling. Metabolite-focused experiments are complementary to microbial co-culture because they isolate concentration, receptor dependence, and downstream signaling, but they do not model microbial production, cross-feeding, colonization, or community adaptation (Bozzetti and Senger, 2022; Ornelas et al., 2022; Feng et al., 2026).

Bile acids illustrate the mechanistic resolution provided by this approach. Microbially modified seven-oxo-DCA and isoDCA produced opposing effects on intestinal tumorigenesis and host signaling (Dong et al., 2024). The bile acid metabolite three-oxo-LCA activated FXR-related signaling, suppressed proliferation in colorectal cancer organoids and intestinal stem cells, and reduced tumor burden in *Apc* ^Min/+^ mice (Sun et al., 2025). The organoid experiment supports a direct epithelial effect, whereas the mouse experiment addresses exposure, metabolism, and tumor outcome. Neither level alone identifies the microbial community that produces the relevant metabolite in an individual patient.

Short-chain fatty acids and tryptophan derivatives also regulate epithelial and immune states. Butyrate influences differentiation, barrier maintenance, and immune signaling, while tryptophan metabolites act through host receptors that differ across epithelial and immune populations (Agus et al., 2018; Ornelas et al., 2022). Organoids can separate epithelial genotype from metabolite exposure and test receptor-dependent signaling. Concentration, exposure route, and the modeled epithelial compartment must nevertheless reflect the conditions under which the metabolite would reach its target.

Metabolite treatment can also clarify observations generated by microbial or dietary studies. Propionate produced by *B. thetaiotaomicron* promoted goblet-cell differentiation (Wang X. et al., 2024), whereas metabolic activity associated with *L. gallinarum*, Coriobacteriaceae, and *L. lactis* HkyuLL 10 was linked to colorectal organoid growth or suppression (Sugimura et al., 2021; Su et al., 2024; Tang et al., 2024). Integrative plasma and fecal metabolomics can nominate candidate mediators during adenoma-colorectal cancer progression, but organoid validation is needed before an association is interpreted as a direct epithelial mechanism (Sun et al., 2024; Shahid, 2025; Kim et al., 2026), as summarized in Figure 1.

**FIGURE 1:**
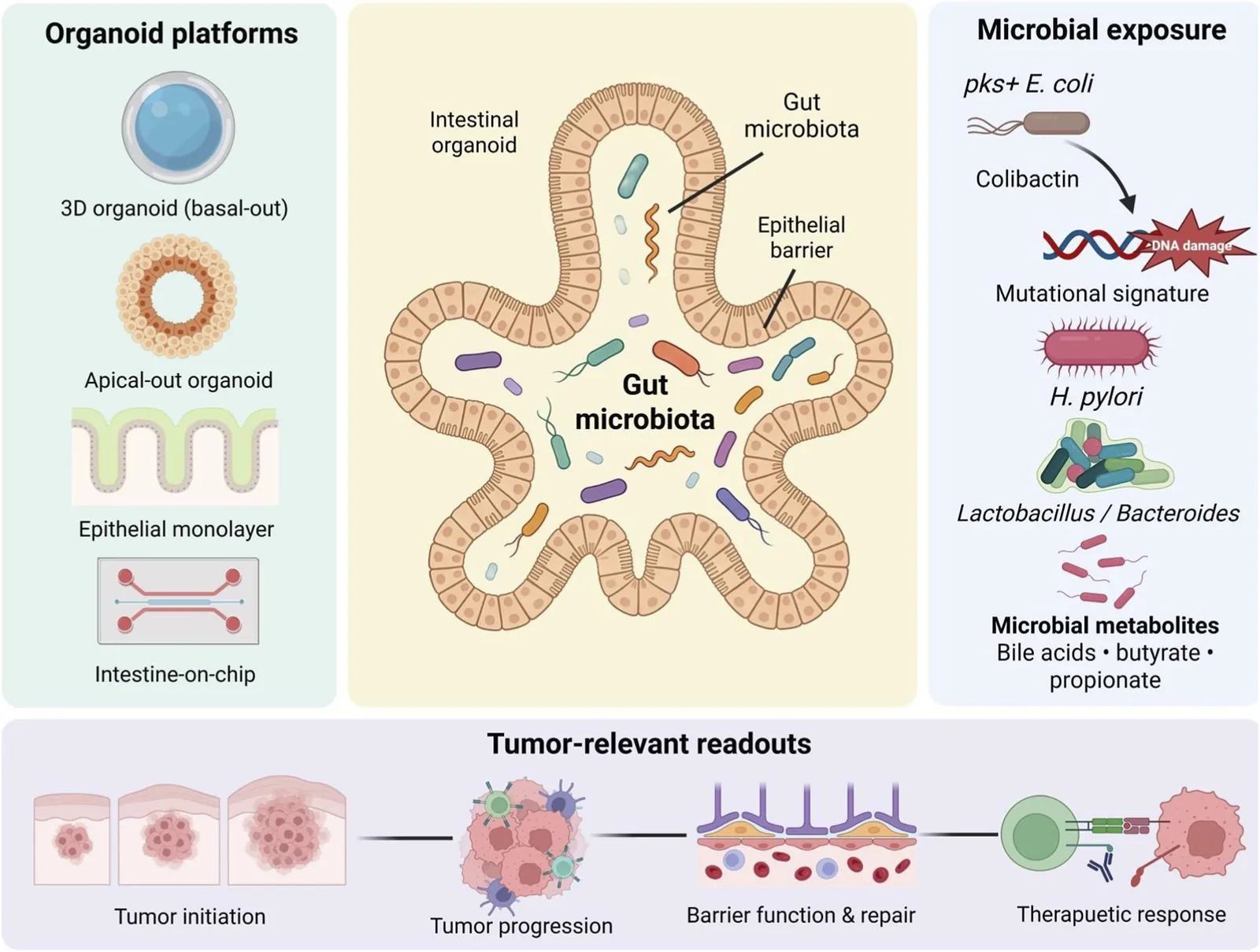
Engineered organoid platforms for mechanistic studies of gastrointestinal microbiota-cancer interactions. The schematic links experimentally accessible organoid architectures to defined microbial exposures and tumor-relevant readouts. Three-dimensional basal-out organoids, apical-out organoids, epithelial monolayers, and intestine-on-chip systems provide different levels of luminal access, directional sampling, barrier measurement, and physicochemical control. Direct organoid-supported examples include colibactin-producing *pks-positive Escherichia coli*, which induces DNA damage and colorectal cancer-associated mutational signatures in human intestinal organoids, and *Helicobacter pylori*, for which direct host-bacterium responses have been demonstrated in human gastric organoid systems. *Lactobacillus, Bacteroides*, and microbial metabolites such as bile acids, butyrate, and propionate illustrate additional exposure classes; depending on the specific mechanism, their cancer- or treatment-related effects may be supported by direct organoid experiments or by complementary animal and clinical evidence. The readout panel summarizes tumor initiation, tumor progression, epithelial barrier function and repair, and therapeutic response without implying that the complete gut microbiota-systemic immunity-distant tumor axis has been reconstructed in a single organoid system.

## 4 Engineering strategies to advance tumor–gut microbiota organoid platforms

### 4.1 Current limitations of tumor–gut microbiota organoid models

The reproducibility of tumor-microbiota organoid experiments depends on both the host and microbial compartments. Host variables include donor, species, tissue region, disease state, genotype, passage, differentiation protocol, extracellular matrix, medium, and epithelial lineage composition. Microbial variables include strain identity, growth phase, inoculum, viability, oxygen tolerance, exposure duration, and whether the organism remains luminal, adherent, invasive, or extracellular (Dutton et al., 2019; Jalili-Firoozinezhad et al., 2019; Carvalho et al., 2023; Monteduro et al., 2023; Taebnia et al., 2023; Srivastava et al., 2024). Reporting only the organoid source and microbial species is insufficient when these conditions determine the phenotype.

Culture materials and passage can change model identity. Intestinal organoids commonly rely on animal-derived basement membrane extract and media containing Wnt, R-spondin, Noggin, EGF, and small-molecule inhibitors. Lot-dependent matrix composition and selective culture conditions can alter differentiation and drug response. Defined hydrogels and scaffold-guided tissues improve control of stiffness, adhesion ligands, and geometry (Foulke-Abel et al., 2016; Gjorevski et al., 2016; Nikolaev et al., 2020), but they still require benchmarking against the tissue state relevant to the hypothesis. Patient-derived tumor organoids may retain clinically important features while undergoing clonal selection or transcriptional drift during long-term propagation (Ben-David et al., 2018; Vlachogiannis et al., 2018; Thorel et al., 2024). Passage range, culture duration, differentiation interval, and contamination testing should therefore be reported at the time of microbial exposure.

Microbial viability is a separate source of variation. Standard organoid media are optimized for mammalian cells rather than bacteria, and residual antibiotics can alter microbial growth. Aerobic culture excludes obligate anaerobes or changes their metabolism, whereas prolonged bacterial expansion may acidify the medium and cause nonspecific epithelial injury. Oxygen-controlled chips address part of this problem (Jalili-Firoozinezhad et al., 2019), but studies must measure oxygen, microbial abundance, community composition, and barrier integrity over time. A stable community cannot be inferred from the starting inoculum.

Incomplete immune reconstruction also limits interpretation. Addition of T cells, macrophages, fibroblasts, or cytokines can test a defined cellular route (Bar-Ephraim et al., 2020; Wang et al., 2025; Najafi et al., 2026; Tsai et al., 2026), but these systems do not reproduce systemic immune trafficking, organ-level pharmacokinetics, or the full tumor ecology. Model complexity should therefore be determined by the causal step under study. Each added compartment requires a measurable function and an appropriate control.

In Sections 4.2–4.5, we distinguish technologies already applied in direct microbiota-organoid experiments from enabling methods that illustrate how molecular, microfluidic, or physicochemical variables can be controlled. Enabling examples are included as methodological precedents and should not be interpreted as evidence for a microbiota-cancer mechanism unless a defined microbial exposure and an organoid-based readout are both present.

### 4.2 Omics-based benchmarking of engineered organoid platforms

High-throughput sequencing and multi-omics methods expand the readouts applied to organoid models. Bulk RNA sequencing can compare an organoid with its source tissue, while single-cell methods can identify loss or expansion of specific epithelial, immune, or stromal populations (Brazovskaja et al., 2019; Liu et al., 2019). Proteomics, epigenomics, and metabolomics can test whether a proposed pathway or microbial product is present. These measurements benchmark or interrogate the model; they do not constitute the physical organoid platform.

Omics-guided studies are most informative when followed by a defined perturbation. In gastric cancer organoids, circ_0008315 was associated with cisplatin resistance, and its knockdown restored drug sensitivity (Fei et al., 2024). In intestinal systems, NIK deficiency altered proliferation and regeneration-related expression, whereas Tcf4 deficiency changed secretory differentiation and antimicrobial peptide production (Morrison et al., 2024; Janeckova et al., 2025). Metagenomic and metabolomic studies can nominate candidate microorganisms or metabolites associated with colorectal cancer (Minot et al., 2024; Sun et al., 2024), but causal interpretation requires testing the candidate exposure in an organoid, animal model, or another functional system. The circ_0008315, NIK, and Tcf4 studies are therefore cited here as examples of omics-guided or genetically defined organoid perturbation, not as direct evidence of microbiota-cancer interactions.

Benchmarking should be hypothesis-specific. A barrier experiment should verify epithelial polarity, lineage composition, permeability, and microbial position. A genotoxicity experiment should measure bacterial burden, DNA damage, mutation, and growth at nonlethal exposure. An immune co-culture should quantify immune-cell persistence, activation, and tumor-cell killing. Large molecular datasets cannot compensate for missing exposure controls or an incorrectly specified tissue model.

### 4.3 Microfluidic and organ-on-chip platforms for microbiota integration

Microfluidic systems are justified when flow, compartmentalization, oxygen control, mechanical deformation, or repeated sampling is part of the biological question. Biopsy-derived human intestinal organoid epithelium has been regenerated in chips that support polarized barrier formation and physiologic transport (Kasendra et al., 2018; Shin et al., 2020). Anaerobic intestine-on-chip systems separate epithelial oxygenation from a low-oxygen luminal compartment and permit prolonged contact with complex microbiota (Jalili-Firoozinezhad et al., 2019). These capabilities address limitations of static organoids, particularly for studies of barrier transport and microbial persistence.

The added control also creates device-specific variation. Channel dimensions, membrane material, extracellular matrix coating, flow rate, shear stress, oxygen permeability, bubble formation, and sampling frequency can alter the result (Dutton et al., 2019; Monteduro et al., 2023; Shetty et al., 2026). Device materials may absorb hydrophobic drugs or metabolites, and small media volumes can amplify evaporation and concentration changes. Physical parameters and chip-only controls should be reported when the material or device can affect the exposure.

Microphysiological systems have also been used to study treatment-related intestinal injury, drug permeability, and epithelial repair (Bouges et al., 2023; AlShmmari et al., 2025; Yan et al., 2025). These models are relevant to cancer when the endpoint is chemotherapy or radiotherapy toxicity, but they should not be presented as direct tumor-microbiota models unless a tumor-associated mechanism or microbial exposure is included. Immune or stromal compartments should likewise be added only when the proposed response depends on those cells (Taebnia et al., 2023; Wang et al., 2025; Najafi et al., 2026).

### 4.4 Engineered microbial communities and hybrid organoid-chip systems

Defined microbial communities occupy an intermediate position between single-strain experiments and fecal inocula. A rationally assembled consortium permits removal and add-back of individual members, measurement of cross-feeding, and control of starting abundance. Gradient hydrogels and microfluidic systems can impose spatial or oxygen structure and have been used to examine microbiome-drug interactions (Zheng et al., 2023). The community must be measured after co-culture because the inoculated ratio may not persist.

Engineered bacteria can provide targeted loss-of-function or gain-of-function tests. Surface expression of the colibactin resistance protein ClbS on engineered bacteria reduced host DNA damage caused by genotoxic colibactin (Yang et al., 2026). This experiment tests the specificity of a proposed microbial mechanism when the engineered strain is compared with an isogenic control and microbial fitness is shown to be equivalent. Delivery platforms without an organoid readout or a microbiota-derived mechanism fall outside the central scope of this review.

Hybrid organoid-chip systems combine organoid-derived human epithelium with controlled transport and microbial exposure (Carvalho et al., 2023; Park et al., 2025). Their value is not the number of technologies combined, but the ability to isolate a transport step, maintain a defined community, or sample a response over time. Clinical translation will require evidence that the added engineering feature improves reproducibility or predicts a patient-relevant outcome.

### 4.5 Physical microenvironmental engineering

Fluid shear stress, oxygen gradients, matrix stiffness, nutrient distribution, and tissue geometry regulate epithelial differentiation and microbial metabolism. Conventional organoids provide limited control over these variables. Defined hydrogels and scaffold-guided tissues control selected matrix and spatial signals (Foulke-Abel et al., 2016; Gjorevski et al., 2016; Nikolaev et al., 2020), while physiodynamic mucosal interface chips support three-dimensional epithelial organization under perfusion (Shin et al., 2020). Gas-modulating systems provide experimental control of local hypoxia (Molley et al., 2023). These approaches are informative only when the resulting tissue is functionally benchmarked. Gas-modulating microcapsules are cited here as an enabling strategy for experimental control of oxygen tension; their direct application to microbiota-cancer organoid systems has not yet been established.

Model maturity and species also affect interpretation. Pluripotent stem cell-derived intestinal tissues, adult tissue-derived enteroids, normal intestinal organoids, and tumor organoids differ in lineage composition, mucus production, metabolism, and developmental state (Sato et al., 2011; Spence et al., 2011; Workman et al., 2017). Mouse organoids support defined genetic perturbation and matched *in vivo* studies, whereas human organoids capture human-specific programs and donor variation. Results should be generalized only to the species and tissue state that were modeled.

Standardization should focus on variables that control the claim rather than impose one platform on all questions. A minimum report should identify species and tissue source, disease state, organoid derivation, architecture and polarity, passage, matrix and medium, microbial preparation and viability, inoculum, exposure route, oxygen condition, duration, host and microbial readouts, and orthogonal validation (Dutton et al., 2019; Carvalho et al., 2023; Taebnia et al., 2023). These principal engineering considerations are summarized in Figure 2.

**FIGURE 2:**
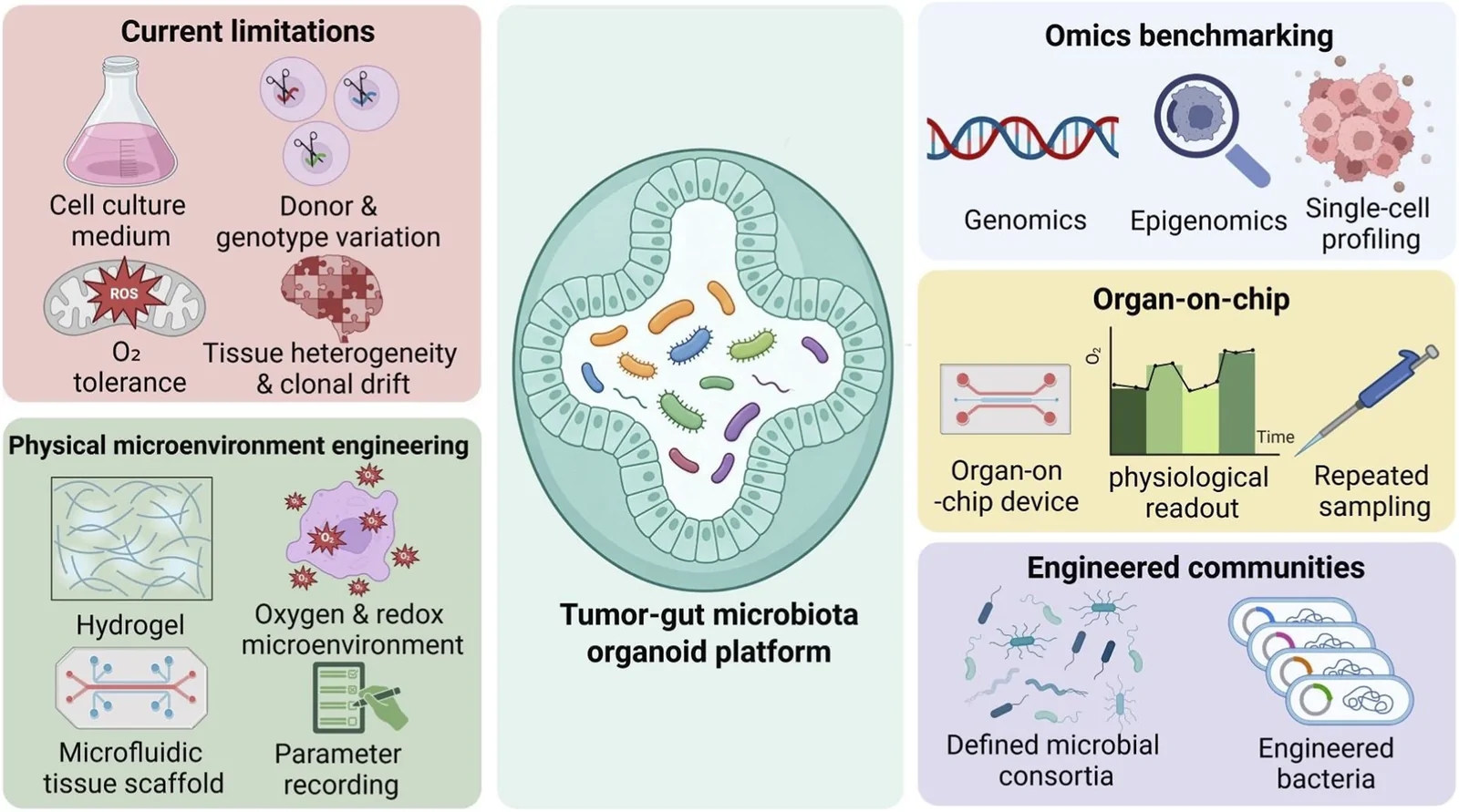
Engineering priorities for tumor-gut microbiota organoid platforms. The central tumor-gut microbiota organoid platform is framed by the principal variables that determine interpretability and reproducibility. Current limitations include culture-medium effects, donor and genotype variation, oxygen tolerance, and tissue heterogeneity or clonal drift. Physical microenvironment engineering uses defined hydrogels, microfluidic scaffolds, oxygen and redox control, and parameter recording to reproduce selected tissue conditions. Omics approaches are shown as benchmarking and analytical readouts rather than structural components of the organoid platform. Organ-on-chip systems add controlled perfusion, physiological measurements, and repeated sampling, whereas defined microbial consortia and engineered bacteria enable controlled microbial perturbation. These modules are enabling strategies unless they are directly linked to a defined microbial exposure and an organoid-based mechanistic readout.

## 5 Conclusions and perspectives

Engineered organoid platforms provide convincing causal evidence for a limited set of microbiota-cancer mechanisms. Repeated exposure of human intestinal organoids to colibactin-producing *E. coli* links a defined bacterium to a colorectal cancer-associated mutational signature (Pleguezuelos-Manzano et al., 2020). Human gastric organoids establish direct epithelial responses to *H. pylori* (McCracken et al., 2014; Holokai et al., 2019). Intestinal organoids and chips resolve barrier injury, repair, polarity-dependent infection, and selected microbial or metabolite effects. These conclusions are strongest when the exposure, host tissue, and readout are experimentally connected.

The principal evidence break occurs when a proposed mechanism requires an intact microbial community, systemic immunity, or a distant tumor. Purified metabolite treatment can establish receptor-level activity but cannot reproduce microbial production or host distribution. A tumor organoid can establish a cancer-cell response but cannot reproduce gut barrier transport or immune priming. Clinical and animal studies support the systemic importance of the gut microbiota, whereas direct human organoid reconstruction of the complete gut microbiota-systemic immunity-distant tumor axis remains limited (Gopalakrishnan et al., 2018; Yao et al., 2026).

The next technical priority is controlled comparability. Microbial viability, oxygen, exposure duration, matrix, medium, passage, epithelial polarity, and immune composition should be treated as experimental determinants. The most useful platform is the least complex system that exposes the relevant surface, preserves the required host compartment, and permits the proposed mediator to be measured and perturbed. This approach will clarify which microbiota-cancer associations reflect direct epithelial mechanisms, which depend on multicellular host responses, and which remain observations requiring functional validation.

## References

[B1] Agus A. Planchais J. Sokol H. (2018). Gut microbiota regulation of tryptophan metabolism in health and disease. Cell Host Microbe 23, 716–724. 10.1016/j.chom.2018.05.003 29902437

[B2] Almeqdadi M. Mana M. D. Roper J. Yilmaz Ö. H. (2019). Gut organoids: mini-tissues in culture to study intestinal physiology and disease. Am. J. Physiol. Cell Physiol. 317, C405–C419. 10.1152/ajpcell.00300.2017 31216420 PMC6766612

[B3] AlShmmari S. K. Fardous R. S. Alhamamah M. A. Cialla-May D. Popp J. Ramadan Q. (2025). Characterization of pro-drug metabolism and drug permeability kinetics in a microphysiological *in vitro* model of the human small intestinal barrier incorporating mucus-generating cells coupled with LC-MS/MS analysis. Adv. Healthc. Mater. 14, e2500692. 10.1002/adhm.202500692 40424025

[B4] Arthur J. C. Perez-Chanona E. Mühlbauer M. Tomkovich S. Uronis J. M. Fan T.-J. (2012). Intestinal inflammation targets cancer-inducing activity of the microbiota. Science 338, 121–123. 10.1126/science.1224820 22903521 PMC3645302

[B5] Asai A. Aihara E. Watson C. Mourya R. Mizuochi T. Shivakumar P. (2017). Paracrine signals regulate human liver organoid maturation from induced pluripotent stem cells. Development 144, 1056–1064. 10.1242/dev.142794 28275009 PMC5358109

[B6] Baas F. S. Brusselaers N. Nagtegaal I. D. Engstrand L. Boleij A. (2024). Navigating beyond associations: opportunities to establish causal relationships between the gut microbiome and colorectal carcinogenesis. Cell Host Microbe 32, 1235–1247. 10.1016/j.chom.2024.07.008 39146796

[B7] Bar-Ephraim Y. E. Kretzschmar K. Clevers H. (2020). Organoids in immunological research. Nat. Rev. Immunol. 20, 279–293. 10.1038/s41577-019-0248-y 31853049

[B8] Bartfeld S. Bayram T. van de Wetering M. Huch M. Begthel H. Kujala P. (2015). *In vitro* expansion of human gastric epithelial stem cells and their responses to bacterial infection. Gastroenterology 148, 126–136.e6. 10.1053/j.gastro.2014.09.042 25307862 PMC4274199

[B9] Ben-David U. Siranosian B. Ha G. Tang H. Oren Y. Hinohara K. (2018). Genetic and transcriptional evolution alters cancer cell line drug response. Nature 560, 325–330. 10.1038/s41586-018-0409-3 30089904 PMC6522222

[B10] Bornholdt J. Müller C. V. Nielsen M. J. Strickertsson J. Rago D. Chen Y. (2023). Detecting host responses to microbial stimulation using primary epithelial organoids. Gut Microbes 15, 2281012. 10.1080/19490976.2023.2281012 37992398 PMC10730191

[B11] Bouges E. Segers C. Leys N. Lebeer S. Zhang J. Mastroleo F. (2023). Human intestinal organoids and microphysiological systems for modeling radiotoxicity and assessing radioprotective agents. Cancers 15, 5859. 10.3390/cancers15245859 38136404 PMC10741417

[B12] Bozzetti V. Senger S. (2022). Organoid technologies for the study of intestinal microbiota-host interactions. Trends Mol. Med. 28, 291–303. 10.1016/j.molmed.2022.02.001 PMC8957533 35232671

[B13] Brazovskaja A. Treutlein B. Camp J. G. (2019). High-throughput single-cell transcriptomics on organoids. Curr. Opin. Biotechnol. 55, 167–171. 10.1016/j.copbio.2018.11.002 30504008

[B14] Carvalho M. R. Yan L.-P. Li B. Zhang C.-H. He Y.-L. Reis R. L. (2023). Gastrointestinal organs and organoids-on-a-chip: advances and translation into the clinics. Biofabrication 15, 042004. 10.1088/1758-5090/acf8fb 37699408

[B15] Cheng M. Xu B. Sun Y. Wang J. Lu Y. Shi C. (2024). ASB3 expression aggravates inflammatory bowel disease by targeting TRAF6 protein stability and affecting the intestinal microbiota. mBio 15, e0204324. 10.1128/mbio.02043-24 39162488 PMC11389410

[B16] Co J. Y. Margalef-Català M. Li X. Mah A. T. Kuo C. J. Monack D. M. (2019). Controlling epithelial polarity: a human enteroid model for host-pathogen interactions. Cell Rep. 26, 2509–2520.e4. 10.1016/j.celrep.2019.01.108 30811997 PMC6391775

[B17] de Visser K. E. Joyce J. A. (2023). The evolving tumor microenvironment: from cancer initiation to metastatic outgrowth. Cancer Cell 41, 374–403. 10.1016/j.ccell.2023.02.016 36917948

[B18] Dong X. Sun F. Secaira-Morocho H. Hui A. Wang K. Cai C. (2024). The dichotomous roles of microbial-modified bile acids 7-oxo-DCA and isoDCA in intestinal tumorigenesis. Proc. Natl. Acad. Sci. U. S. A. 121, e2317596121. 10.1073/pnas.2317596121 39531490 PMC11588130

[B19] Drost J. Clevers H. (2018). Organoids in cancer research. Nat. Rev. Cancer 18, 407–418. 10.1038/s41568-018-0007-6 29692415

[B20] Dutton J. S. Hinman S. S. Kim R. Wang Y. Allbritton N. L. (2019). Primary cell-derived intestinal models: recapitulating physiology. Trends Biotechnol. 37, 744–760. 10.1016/j.tibtech.2018.12.001 30591184 PMC6571163

[B21] Fatehullah A. Tan S. H. Barker N. (2016). Organoids as an *in vitro* model of human development and disease. Nat. Cell Biol. 18, 246–254. 10.1038/ncb3312 26911908

[B22] Fei Y. Cao D. Li Y. Wang Z. Dong R. Zhu M. (2024). Circ_0008315 promotes tumorigenesis and cisplatin resistance and acts as a nanotherapeutic target in gastric cancer. J. Nanobiotechnol. 22, 519. 10.1186/s12951-024-02760-6 PMC11360491 39210348

[B23] Feng C. He Q. Kwok L.-Y. Sun Z. (2026). Gut microbiota-derived metabolites as systemic messengers: mechanisms of gut-organ communication and translational opportunities. Trends Food Sci. Technol. 174, 105818. 10.1016/j.tifs.2026.105818

[B24] Fischer J. C. Göttert S. Giller M. Heinrich P. Fan K. Khalid O. (2025). Tissue-adapted tregs harness inflammatory signals to promote intestinal repair from therapy-related injury. Signal Transduct. Target. Ther. 10, 384. 10.1038/s41392-025-02476-5 41290566 PMC12647259

[B25] Foulke-Abel J. In J. Yin J. Zachos N. C. Kovbasnjuk O. Estes M. K. (2016). Human enteroids as a model of upper small intestinal ion transport physiology and pathophysiology. Gastroenterology 150, 638–649.e8. 10.1053/j.gastro.2015.11.047 26677983 PMC4766025

[B26] Gao J. Xiong T. Grabauskas G. Owyang C. (2022). Mucosal serotonin reuptake transporter expression in irritable bowel syndrome is modulated by gut microbiota *via* mast cell-prostaglandin E2. Gastroenterology 162, 1962–1974.e6. 10.1053/j.gastro.2022.02.016 35167867 PMC9117493

[B27] Gjorevski N. Sachs N. Manfrin A. Giger S. Bragina M. E. Ordóñez-Morán P. (2016). Designer matrices for intestinal stem cell and organoid culture. Nature 539, 561–564. 10.1038/nature20168 27851739

[B28] Gopalakrishnan V. Spencer C. N. Nezi L. Reuben A. Andrews M. C. Karpinets T. V. (2018). Gut microbiome modulates response to anti-PD-1 immunotherapy in melanoma patients. Science 359, 97–103. 10.1126/science.aan4236 29097493 PMC5827966

[B29] Helmink B. A. Khan M. A. W. Hermann A. Gopalakrishnan V. Wargo J. A. (2019). The microbiome, cancer, and cancer therapy. Nat. Med. 25, 377–388. 10.1038/s41591-019-0377-7 30842679

[B30] Hill D. R. Huang S. Nagy M. S. Yadagiri V. K. Fields C. Mukherjee D. (2017). Bacterial colonization stimulates a complex physiological response in the immature human intestinal epithelium. eLife 6, e29132. 10.7554/eLife.29132 29110754 PMC5711377

[B31] Holokai L. Chakrabarti J. Broda T. Chang J. Hawkins J. A. Sundaram N. (2019). Increased programmed death-ligand 1 is an early epithelial cell response to helicobacter pylori infection. PLOS Pathog. 15, e1007468. 10.1371/journal.ppat.1007468 30703170 PMC6380601

[B32] Huch M. Gehart H. van Boxtel R. Hamer K. Blokzijl F. Verstegen M. M. A. (2015). Long-term culture of genome-stable bipotent stem cells from adult human liver. Cell 160, 299–312. 10.1016/j.cell.2014.11.050 25533785 PMC4313365

[B33] Jalili-Firoozinezhad S. Gazzaniga F. S. Calamari E. L. Camacho D. M. Fadel C. W. Bein A. (2019). A complex human gut microbiome cultured in an anaerobic intestine-on-a-chip. Nat. Biomed. Eng. 3, 521–531. 10.1038/s41551-019-0397-0 31086325 PMC6658209

[B34] Janeckova L. Stastna M. Hrckulak D. Berkova L. Kubovciak J. Onhajzer J. (2025). Tcf4 regulates secretory cell fate decisions in the small intestine and colon tumors: insights from transcriptomic, histological, and microbiome analyses. Stem Cell Res. Ther. 16, 170. 10.1186/s13287-025-04280-y 40221753 PMC11993999

[B35] Kasendra M. Tovaglieri A. Sontheimer-Phelps A. Jalili-Firoozinezhad S. Bein A. Chalkiadaki A. (2018). Development of a primary human small intestine-on-a-chip using biopsy-derived organoids. Sci. Rep. 8, 2871. 10.1038/s41598-018-21201-7 29440725 PMC5811607

[B36] Kim H. J. Huh D. Hamilton G. Ingber D. E. (2012). Human gut-on-a-chip inhabited by microbial flora that experiences intestinal peristalsis-like motions and flow. Lab. Chip 12, 2165–2174. 10.1039/c2lc40074j 22434367

[B37] Kim K.-H. Yoo M. Choi M. Y. Yoo B. C. (2026). A practical roadmap for clinical translation of metabolic biomarkers: a review. Int. J. Mol. Sci. 27, 2030. 10.3390/ijms27042030 41752166 PMC12940984

[B38] Kozuka K. He Y. Koo-McCoy S. Kumaraswamy P. Nie B. Shaw K. (2017). Development and characterization of a human and mouse intestinal epithelial cell monolayer platform. Stem Cell Rep. 9, 1976–1990. 10.1016/j.stemcr.2017.10.013 PMC5785676 29153987

[B39] Li B. Tang Y. Huang Z. Ma L. Song J. Xue L. (2026). Synergistic innovation in organ-on-a-chip and organoid technologies: reshaping the future of disease modeling, drug development, and precision medicine. Protein Cell 17, 686–703. 10.1093/procel/pwaf058 40653861 PMC13429468

[B40] Lindemans C. A. Calafiore M. Mertelsmann A. M. O’Connor M. H. Dudakov J. A. Jenq R. R. (2015). Interleukin-22 promotes intestinal-stem-cell-mediated epithelial regeneration. Nature 528, 561–564. 10.1038/nature16460 26649819 PMC4720437

[B41] Liu Y. Mi Y. Mueller T. Kreibich S. Williams E. G. Van Drogen A. (2019). Multi-omic measurements of heterogeneity in HeLa cells across laboratories. Nat. Biotechnol. 37, 314–322. 10.1038/s41587-019-0037-y 30778230

[B42] Liu X. Zhou Z. Zhang Y. Zhong H. Cai X. Guan R. (2025). Recent progress on the organoids: techniques, advantages and applications. Biomed. Pharmacother. = Biomed. Pharmacother. 185, 117942. 10.1016/j.biopha.2025.117942 40043462

[B43] Lu H. Ye C. Liu J. Rabson A. B. Verzi M. De S. (2021). Requirement of bccip for the regeneration of intestinal progenitors. Am. J. Pathol. 191, 66–78. 10.1016/j.ajpath.2020.09.009 33039352 PMC7857062

[B44] Mao Y. Hu H. (2024). Establishment of advanced tumor organoids with emerging innovative technologies. Cancer Lett. 598, 217122. 10.1016/j.canlet.2024.217122 39029781

[B45] McCauley H. A. Wells J. M. (2017). Pluripotent stem cell-derived organoids: using principles of developmental biology to grow human tissues in a dish. Development 144, 958–962. 10.1242/dev.140731 28292841 PMC5358106

[B46] McCracken K. W. Catá E. M. Crawford C. M. Sinagoga K. L. Schumacher M. Rockich B. E. (2014). Modelling human development and disease in pluripotent stem-cell-derived gastric organoids. Nature 516, 401–404. 10.1038/nature13863 25363776 PMC4270898

[B47] Minot S. S. Li N. Srinivasan H. Ayers J. L. Yu M. Koester S. T. (2024). Colorectal cancer-associated bacteria are broadly distributed in global microbiomes and drivers of precancerous change. Sci. Rep. 14, 23646. 10.1038/s41598-024-70702-1 39384807 PMC11464690

[B48] Miyakawa Y. Otsuka M. Shibata C. Seimiya T. Yamamoto K. Ishibashi R. (2024). Gut bacteria-derived membrane vesicles induce colonic dysplasia by inducing DNA damage in colon epithelial cells. Cell. Mol. Gastroenterol. Hepatol. 17, 745–767. 10.1016/j.jcmgh.2024.01.010 38309455 PMC10966291

[B49] Molley T. G. Jiang S. Ong L. Kopecky C. Ranaweera C. D. Jalandhra G. K. (2023). Gas-modulating microcapsules for spatiotemporal control of hypoxia. Proc. Natl. Acad. Sci. U. S. A. 120, e2217557120. 10.1073/pnas.2217557120 37040415 PMC10120079

[B50] Mommersteeg M. C. Simovic I. Yu B. van Nieuwenburg S. a. V. Bruno I. M. J. Doukas M. (2022). Autophagy mediates ER stress and inflammation in helicobacter pylori-related gastric cancer. Gut Microbes 14, 2015238. 10.1080/19490976.2021.2015238 34965181 PMC8726742

[B51] Monteduro A. G. Rizzato S. Caragnano G. Trapani A. Giannelli G. Maruccio G. (2023). Organs-on-chips technologies - a guide from disease models to opportunities for drug development. Biosens. Bioelectron. 231, 115271. 10.1016/j.bios.2023.115271 37060819

[B52] Morrison H. A. Eden K. Trusiano B. Rothschild D. E. Qin Y. Wade P. A. (2024). NF-κB inducing kinase attenuates colorectal cancer by regulating noncanonical NF-κB mediated colonic epithelial cell regeneration. Cell. Mol. Gastroenterol. Hepatol. 18, 101356. 10.1016/j.jcmgh.2024.05.004 38750899 PMC11278896

[B53] Mucciolo G. Araos Henríquez J. Jihad M. Pinto Teles S. Manansala J. S. Li W. (2024). EGFR-activated myofibroblasts promote metastasis of pancreatic cancer. Cancer Cell 42, 101–118.e11. 10.1016/j.ccell.2023.12.002 38157863

[B54] Najafi F. Zoghi N. A. Khalili H. Najafi N. Yazdi M. H. Sisakht M. M. (2026). Organoid-immune Co-cultures: a next-generation approach to disease modeling. Mol. Biol. Rep. 53, 360. 10.1007/s11033-026-11450-5 41636909

[B55] Neal J. T. Li X. Zhu J. Giangarra V. Grzeskowiak C. L. Ju J. (2018). Organoid modeling of the tumor immune microenvironment. Cell 175, 1972–1988.e16. 10.1016/j.cell.2018.11.021 30550791 PMC6656687

[B56] Nikolaev M. Mitrofanova O. Broguiere N. Geraldo S. Dutta D. Tabata Y. (2020). Homeostatic mini-intestines through scaffold-guided organoid morphogenesis. Nature 585, 574–578. 10.1038/s41586-020-2724-8 32939089

[B57] Öhlund D. Handly-Santana A. Biffi G. Elyada E. Almeida A. S. Ponz-Sarvise M. (2017). Distinct populations of inflammatory fibroblasts and myofibroblasts in pancreatic cancer. J. Exp. Med. 214, 579–596. 10.1084/jem.20162024 28232471 PMC5339682

[B58] Ornelas A. Dowdell A. S. Lee J. S. Colgan S. P. (2022). Microbial metabolite regulation of epithelial cell-cell interactions and barrier function. Cells 11, 944. 10.3390/cells11060944 35326394 PMC8946845

[B59] Park B. Park J. Han S. Kwon T. Sung J. Y. Kim J. (2025). Advances in organoid-on-a-chip for recapitulation of human physiological events. Mater. Today 84, 75–94. 10.1016/j.mattod.2025.02.002

[B60] Pleguezuelos-Manzano C. Puschhof J. Rosendahl Huber A. van Hoeck A. Wood H. M. Nomburg J. (2020). Mutational signature in colorectal cancer caused by genotoxic pks+ E. coli. Nature 580, 269–273. 10.1038/s41586-020-2080-8 32106218 PMC8142898

[B61] Rauner G. Gupta P. B. Kuperwasser C. (2025). From 2D to 3D and beyond: the evolution and impact of *in vitro* tumor models in cancer research. Nat. Methods 22, 1776–1787. 10.1038/s41592-025-02769-1 40715728

[B62] Rosendahl Huber A. Pleguezuelos-Manzano C. Puschhof J. (2021). A bacterial mutational footprint in colorectal cancer genomes. Br. J. Cancer 124, 1751–1753. 10.1038/s41416-021-01273-5 33742142 PMC8144397

[B63] Sato T. Vries R. G. Snippert H. J. van de Wetering M. Barker N. Stange D. E. (2009). Single Lgr5 stem cells build crypt-villus structures *in vitro* without a mesenchymal niche. Nature 459, 262–265. 10.1038/nature07935 19329995

[B64] Sato T. Stange D. E. Ferrante M. Vries R. G. J. Van Es J. H. Van den Brink S. (2011). Long-term expansion of epithelial organoids from human colon, adenoma, adenocarcinoma, and barrett’s epithelium. Gastroenterology 141, 1762–1772. 10.1053/j.gastro.2011.07.050 21889923

[B65] Shahid U. (2025). Microbiome-guided precision medicine: mechanistic insights, multi-omics integration, and translational horizons. J. Precis. Med. Health Dis. 3, 100018. 10.1016/j.premed.2025.100018

[B66] Shetty S. Kumar N. A. N. Kabekkodu S. P. Chakrabarty S. (2026). A comprehensive review of microfluidic-based organ-on-chip technology in the application of preclinical drug discovery studies. Biochim. Biophys. Acta, Rev. Cancer 1881, 189627. 10.1016/j.bbcan.2026.189627 42235834

[B67] Shin Y. C. Shin W. Koh D. Wu A. Ambrosini Y. M. Min S. (2020). Three-dimensional regeneration of patient-derived intestinal organoid epithelium in a physiodynamic mucosal interface-on-a-chip. Micromachines 11, 663. 10.3390/mi11070663 32645991 PMC7408321

[B68] Sigal M. Logan C. Y. Kapalczynska M. Mollenkopf H.-J. Berger H. Wiedenmann B. (2017). Stromal R-spondin orchestrates gastric epithelial stem cells and gland homeostasis. Nature 548, 451–455. 10.1038/nature23642 28813421

[B69] Spence J. R. Mayhew C. N. Rankin S. A. Kuhar M. F. Vallance J. E. Tolle K. (2011). Directed differentiation of human pluripotent stem cells into intestinal tissue *in vitro* . Nature 470, 105–109. 10.1038/nature09691 21151107 PMC3033971

[B70] Srivastava S. K. Foo G. W. Aggarwal N. Chang M. W. (2024). Organ-on-chip technology: opportunities and challenges. Biotechnol. Notes 5, 8–12. 10.1016/j.biotno.2024.01.001 39416695 PMC11446384

[B71] Su A. C. Y. Ding X. Lau H. C. H. Kang X. Li Q. Wang X. (2024). Lactococcus lactis HkyuLL 10 suppresses colorectal tumourigenesis and restores gut microbiota through its generated alpha-mannosidase. Gut 73, 1478–1488. 10.1136/gutjnl-2023-330835 38599786 PMC11347254

[B72] Suarez G. Romero-Gallo J. Piazuelo M. B. Sierra J. C. Delgado A. G. Washington M. K. (2019). Nod1 imprints inflammatory and carcinogenic responses toward the gastric pathogen helicobacter pylori. Cancer Res. 79, 1601–1611. 10.1158/0008-5472.CAN-18-2651 30696658 PMC6445772

[B73] Sugimura N. Li Q. Chu E. S. H. Lau H. C. H. Fong W. Liu W. (2021). Lactobacillus gallinarum modulates the gut microbiota and produces anti-cancer metabolites to protect against colorectal tumourigenesis. Gut 71, 2011–2021. 10.1136/gutjnl-2020-323951 34937766 PMC9484392

[B74] Sun Y. Zhang X. Hang D. Lau H. C.-H. Du J. Liu C. (2024). Integrative plasma and fecal metabolomics identify functional metabolites in adenoma-colorectal cancer progression and as early diagnostic biomarkers. Cancer Cell 42, 1386–1400.e8. 10.1016/j.ccell.2024.07.005 39137727

[B75] Sun F. Wang K. Dong X. Secaira-Morocho H. Hui A. Cai C. (2025). The microbial bile acid metabolite 3-oxo-LCA inhibits colorectal cancer progression. Cancer Res. 85, 4937–4957. 10.1158/0008-5472.CAN-24-3898 PMC12871761 40960502

[B76] Taebnia N. Römling U. Lauschke V. M. (2023). *In vitro* and *ex vivo* modeling of enteric bacterial infections. Gut Microbes 15, 2158034. 10.1080/19490976.2022.2158034 36576310 PMC9809952

[B77] Tang Q. Huang H. Xu H. Xia H. Zhang C. Ye D. (2024). Endogenous coriobacteriaceae enriched by a high-fat diet promotes colorectal tumorigenesis through the CPT1A-ERK axis. Npj Biofilms Microbiomes 10, 5. 10.1038/s41522-023-00472-7 38245554 PMC10799938

[B78] Thorel L. Perréard M. Florent R. Divoux J. Coffy S. Vincent A. (2024). Patient-derived tumor organoids: a new avenue for preclinical research and precision medicine in oncology. Exp. Mol. Med. 56, 1531–1551. 10.1038/s12276-024-01272-5 38945959 PMC11297165

[B79] Tiriac H. Belleau P. Engle D. D. Plenker D. Deschênes A. Somerville T. D. D. (2018). Organoid profiling identifies common responders to chemotherapy in pancreatic cancer. Cancer Discov. 8, 1112–1129. 10.1158/2159-8290.CD-18-0349 29853643 PMC6125219

[B80] Tsai M.-T. Callaghan R. Ng C. Peres-Tintin L. Matthews D. B. Stanford N. (2026). Intestinal epithelial TLR5 signaling promotes barrier-supportive macrophages. Sci. Immunol. 11, eadr4057. 10.1126/sciimmunol.adr4057 41544147 PMC13016028

[B81] Vazquez-Armendariz A. I. Tata P. R. (2023). Recent advances in lung organoid development and applications in disease modeling. J. Clin. Invest. 133, e170500. 10.1172/JCI170500 37966116 PMC10645385

[B82] Vlachogiannis G. Hedayat S. Vatsiou A. Jamin Y. Fernández-Mateos J. Khan K. (2018). Patient-derived organoids model treatment response of metastatic gastrointestinal cancers. Science 359, 921–926. 10.1126/science.aao2774 29472484 PMC6112415

[B83] Wang Q. Guo F. Zhang Q. Hu T. Jin Y. Yang Y. (2024a). Organoids in gastrointestinal diseases: from bench to clinic. MedComm 5, e574. 10.1002/mco2.574 PMC11214594 38948115

[B84] Wang X. Cai Z. Wang Q. Wu C. Sun Y. Wang Z. (2024b). Bacteroides methylmalonyl-CoA mutase produces propionate that promotes intestinal goblet cell differentiation and homeostasis. Cell Host Microbe 32, 63–78.e7. 10.1016/j.chom.2023.11.005 38056459

[B85] Wang J. Tao X. Zhu J. Dai Z. Du Y. Xie Y. (2025). Tumor organoid-immune co-culture models: exploring a new perspective of tumor immunity. Cell Death Discov. 11, 195. 10.1038/s41420-025-02407-x 40268893 PMC12019369

[B86] Wang D. Villenave R. Stokar-Regenscheit N. Clevers H. (2026). Human organoids as 3D *in vitro* platforms for drug discovery: opportunities and challenges. Nat. Rev. Drug Discov. 25, 204–226. 10.1038/s41573-025-01317-y 41225057

[B87] Workman M. J. Mahe M. M. Trisno S. Poling H. M. Watson C. L. Sundaram N. (2017). Engineered human pluripotent-stem-cell-derived intestinal tissues with a functional enteric nervous system. Nat. Med. 23, 49–59. 10.1038/nm.4233 27869805 PMC5562951

[B88] Wu H. Xie S. Miao J. Li Y. Wang Z. Wang M. (2020). Lactobacillus reuteri maintains intestinal epithelial regeneration and repairs damaged intestinal mucosa. Gut Microbes 11, 997–1014. 10.1080/19490976.2020.1734423 32138622 PMC7524370

[B89] Yan X. Lin X. Wu J. Zheng L. Liu Y. Wu F. (2025). Mitigation of chemotherapy-induced gut dysbiosis and diarrhea by supplementation with heat-killed bacteroides fragilis. BMC Med. 23, 408. 10.1186/s12916-025-04233-5 40624638 PMC12235877

[B90] Yang S. Wang Z. Fang C. Yang M. Khawaled S. Bonanno S. (2026). Surface expression of antitoxin on engineered bacteria neutralizes genotoxic colibactin in the gut. Nat. Microbiol. 11, 53–66. 10.1038/s41564-025-02177-3 41361522 PMC12833714

[B91] Yao Y. Zhu Y. Chen K. Chen J. Li Y. Li D. (2026). Microbiota in cancer: current understandings and future perspectives. Signal Transduct. Target. Ther. 11, 39. 10.1038/s41392-025-02335-3 41629271 PMC12864941

[B92] Zheng D.-W. Qiao J.-Y. Ma J.-C. An J.-X. Yang C.-H. Zhang Y. (2023). A microbial community cultured in gradient hydrogel for investigating gut microbiome-drug interaction and guiding therapeutic decisions. Adv. Mater. 35, e2300977. 10.1002/adma.202300977 37029611

